# Spotlight influenza: Extending influenza surveillance to detect non-influenza respiratory viruses of public health relevance: analysis of surveillance data, Belgium, 2015 to 2019

**DOI:** 10.2807/1560-7917.ES.2021.26.38.2001104

**Published:** 2021-09-23

**Authors:** Lorenzo Subissi, Nathalie Bossuyt, Marijke Reynders, Michèle Gérard, Nicolas Dauby, Patrick Lacor, Siel Daelemans, Bénédicte Lissoir, Xavier Holemans, Koen Magerman, Door Jouck, Marc Bourgeois, Bénédicte Delaere, Sophie Quoilin, Steven Van Gucht, Isabelle Thomas, Cyril Barbezange

**Affiliations:** 1 National Influenza Centre, Sciensano, Brussels, Belgium; 2 European Public Health Microbiology Training Programme (EUPHEM), European Centre for Disease Prevention and Control, Stockholm, Sweden; 3 Epidemiology of Infectious Diseases, Sciensano, Brussels, Belgium; 4 Department of Laboratory Medicine, Medical Microbiology, Algemeen Ziekenhuis Sint-Jan, Brugge-Oostende AV, Belgium; 5 Centre Hospitalier Universitaire St-Pierre, Brussels, Belgium; 6 Centre for Environmental Health and Occupational Health, School of Public Health, Université Libre de Bruxelles (ULB), Brussels, Belgium; 7 Internal Medicine-Infectious Diseases, Universitair Ziekenhuis Brussel, Brussels, Belgium; 8 Pediatric Pulmonary and Infectious Diseases, Universitair Ziekenhuis Brussel, Brussels, Belgium; 9 Microbiology, Grand Hôpital de Charleroi, Charleroi, Belgium; 10 Infectiology, Grand Hôpital de Charleroi, Charleroi, Belgium; 11 Clinical Laboratory, Jessa Ziekenhuis, Hasselt, Belgium; 12 Infection Control, Jessa Ziekenhuis, Hasselt, Belgium; 13 Centre Hospitalier Universitaire UCL Namur, Ysoir, Belgium

**Keywords:** severe acute respiratory infection, influenza-like illness, human metapneumovirus, coronavirus, respiratory syncytial virus

## Abstract

**Background:**

Seasonal influenza-like illness (ILI) affects millions of people yearly. Severe acute respiratory infections (SARI), mainly influenza, are a leading cause of hospitalisation and mortality. Increasing evidence indicates that non-influenza respiratory viruses (NIRV) also contribute to the burden of SARI. In Belgium, SARI surveillance by a network of sentinel hospitals has been ongoing since 2011.

**Aim:**

We report the results of using in-house multiplex qPCR for the detection of a flexible panel of viruses in respiratory ILI and SARI samples and the estimated incidence rates of SARI associated with each virus.

**Methods:**

We defined ILI as an illness with onset of fever and cough or dyspnoea. SARI was defined as an illness requiring hospitalisation with onset of fever and cough or dyspnoea within the previous 10 days. Samples were collected in four winter seasons and tested by multiplex qPCR for influenza virus and NIRV. Using catchment population estimates, we calculated incidence rates of SARI associated with each virus.

**Results:**

One third of the SARI cases were positive for NIRV, reaching 49.4% among children younger than 15 years. In children younger than 5 years, incidence rates of NIRV-associated SARI were twice that of influenza (103.5 vs 57.6/100,000 person-months); co-infections with several NIRV, respiratory syncytial viruses, human metapneumoviruses and picornaviruses contributed most (33.1, 13.6, 15.8 and 18.2/100,000 person-months, respectively).

**Conclusion:**

Early testing for NIRV could be beneficial to clinical management of SARI patients, especially in children younger than 5 years, for whom the burden of NIRV-associated disease exceeds that of influenza.

## Introduction

Acute viral infections of the respiratory tract are common in humans. According to the World Health Organization (WHO), complications such as lower respiratory tract infections and pneumonia are among the main causes of mortality in children and elderly people worldwide [1]. The burden attributed to seasonal influenza virus has long received most of the attention [2], but the involvement of non-influenza respiratory viruses (NIRV), such as respiratory syncytial virus and human metapneumovirus, is increasingly documented [3,4]. However, the burden of NIRV [5-7] compared with seasonal influenza [8] is not sufficiently estimated [9]. These data would allow to better understand the need for enhanced surveillance of these viruses during winter seasons with the aim of improving patient management.

The WHO implemented the influenza surveillance network in 1952 to monitor the continuous antigenic changes of the virus and to guide vaccine composition. Following the 2009 influenza pandemic, the scope of the surveillance was extended to better assess the severity and burden of influenza viruses. In Belgium, influenza virus surveillance is organised by the national public health institute, Sciensano, which also hosts the National Influenza Centre (NIC). Two sentinel networks are in place: (i) surveillance of influenza-like illness (ILI) in the community, based on general practitioners (GPs) providing information on mild cases (in place since the mid-1970s) and (ii) surveillance of severe acute respiratory infection (SARI) based on six sentinel hospitals providing information on hospitalised patients (set up after the 2009 influenza pandemic and operating every season since 2011). Starting with the 2015/16 influenza season, the NIC introduced systematic testing of NIRV by qPCR in addition to testing and characterising influenza viruses. We report here the results of systematic NIRV testing of ILI and SARI surveillance samples for four seasons (2015/16 to 2018/19) and estimate the burden and severity of NIRV compared with influenza.

## Methods

### Settings, participants and variables

Sentinel-based ILI surveillance was performed through a network of GPs widely distributed throughout Belgium, representing ca 1.5% of all Belgian GPs and covering more than 1.1% of the population. Patients were enrolled during each influenza season surveillance period (from week 40 to week 20). Recommendations were that nasopharyngeal swabs were obtained weekly from the first two ILI patients belonging to two different households. SARI patients were recruited through the sentinel network of six hospitals in Belgium, two in each administrative region of the country: Flanders, Wallonia and Brussels-Capital. It was recommended that paediatric and adult units systematically collect detailed clinico-epidemiological data and a respiratory specimen from all patients meeting the SARI case definition during the epidemic period of seasonal influenza (starting and ending weeks between January and April, depending on influenza activity). 

Based on WHO guidelines [10], ILI and SARI cases were defined as acute respiratory illness with onset within the last 10 days, with measured or reported fever of ≥ 38 °C and with cough and/or dyspnoea. In addition, overnight hospitalisation was a required criterion for SARI cases. Patients who did not give informed consent (either directly or through a parent or legal guardian) were not enrolled in the study nor sampled. 

Standardised questionnaires were used to collect data on age, sex, clinical signs included in the case definition, status of vaccination against influenza viruses, administration of a neuraminidase inhibitor antiviral and/or antibiotic treatment, and co-morbidities known as risk factors of severity. Follow-up data during hospitalisation were also reported for the SARI cases to evaluate disease severity and included the detection of pneumonia based on chest radiography, and/or the development of acute respiratory distress syndrome, and/or the requirement for respiratory assistance and/or for extracorporeal membrane oxygenation, and/or the admission to an intensive care unit, and/or death (all-cause death).

### Laboratory testing

Respiratory samples were analysed at the Belgian National Influenza Centre. Viral nucleic acids were extracted using NucliSENS EasyMag (BioMerieux Benelux, Brussels, Belgium). The following respiratory virus targets were detected using several in-house multiplex qPCRs: influenza virus types A and B (and subsequent subtype/lineage), respiratory syncytial virus types A and B, human metapneumoviruses, parainfluenzavirus types 1, 2, 3 and 4, coronaviruses CoV-OC43, CoV-NL63 and CoV-229E, adenoviruses, picornaviruses (*Rhinovirus* and *Enterovirus* genera), specific enterovirus D68, parechovirus and bocavirus as previously described [11].

### Incidence rates of virus-associated severe acute respiratory infection 

To evaluate some aspects of the burden of disease of virus-associated SARI, the catchment population estimates from 2017 (mid-year of the study period) were used to calculate monthly incidence rate per 100,000 population during the winter season, based on WHO recommendations [10]. Catchment populations of the SARI hospitals by age group were estimated based on hospital admission data (i.e. the proportion of the different municipalities) and the total population of these municipalities; this rough estimation has the advantage that it is easily extracted without extra workload for the hospitals. The number of months covered by the study period (i.e. 14.9 months), used for the denominator, was calculated by adding up the number of weeks with active SARI surveillance for each season (i.e. 64 weeks), multiplied by 7 days and divided by the average number of days in a month during the winter period (December to April, i.e. 30 days). Age group-specific catchment populations of the six hospitals were summed up to calculate the monthly incidence rate by age group (< 5, 5–14, 15–64 and ≥ 65 years) for virus-associated SARI. The 95% confidence intervals (CI) were calculated using the Rothman–Greenland method [12].

### Ethical statement

The SARI surveillance protocol was approved by a central Ethical Committee (reference AK/12–02–11/4111; in 2011: Centre Hospitalier Universitaire St-Pierre, Brussels, Belgium; from 2014 onwards: Universitair Ziekenhuis Brussel, Brussels, Belgium) and the local ethical committees of each participating hospital. Informed consent was obtained from all participants or parents/guardians.

## Results

### Study population

**Figure 1:**
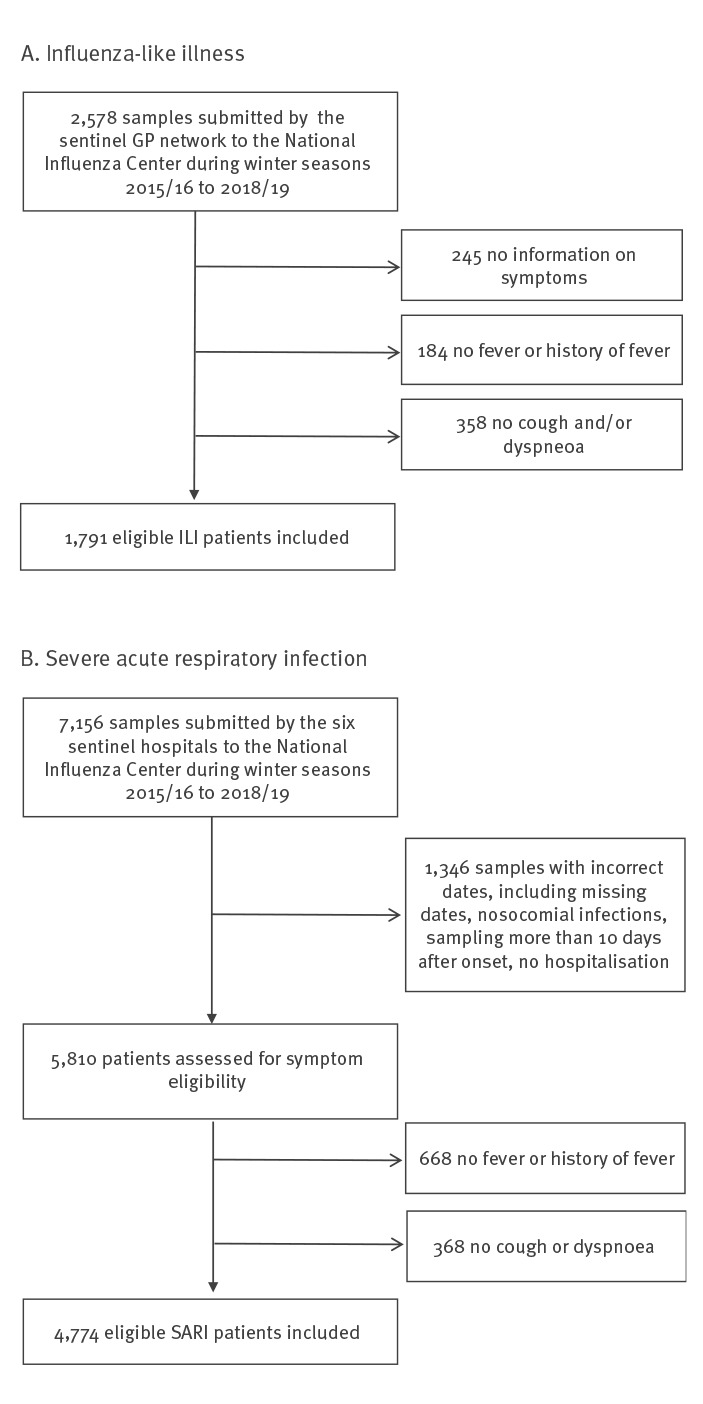
Flow diagram for participant inclusion in the study for surveillance of influenza-like illness and severe acute respiratory infections, Belgium, 2015–2019 (n = 9,734)

GP: general practitioner; ILI: influenza-like illness; SARI: severe acute respiratory infection.

Over the four seasons under study 1,791 ILI (Figure 1A) and 4,774 SARI (Figure 1B) patients responding to the case definition were retained in the analysis. Among the ILI patients with known age, 78.7% were adults (15–64 years-old, 1,356/1,724), 14.2% were children (< 15 years-old, 244/1,724) and 7.2% were older adults (65 years and older, 124/1,724). Half were female (833/1,692), including 11 who were pregnant. Among the SARI patients, there were slightly more male than female patients (52.2%; 2,428/4,650), and 28 women were pregnant. Children and older adults represented, respectively, 35.5% (1,689/4,762) and 44.1% (2,099/4,762) of the SARI cases with known age. Among children, 44.6% and 42.1% were younger than 1 year (753/1,689) and between 1 and 4 years-old (711/1,689), respectively. The six sentinel hospitals contributed heterogeneously to the distribution of SARI patients in terms of age, with one hospital reporting more paediatric cases (Supplementary Table S1).

### Overall respiratory virus detection rates

**Figure 2:**
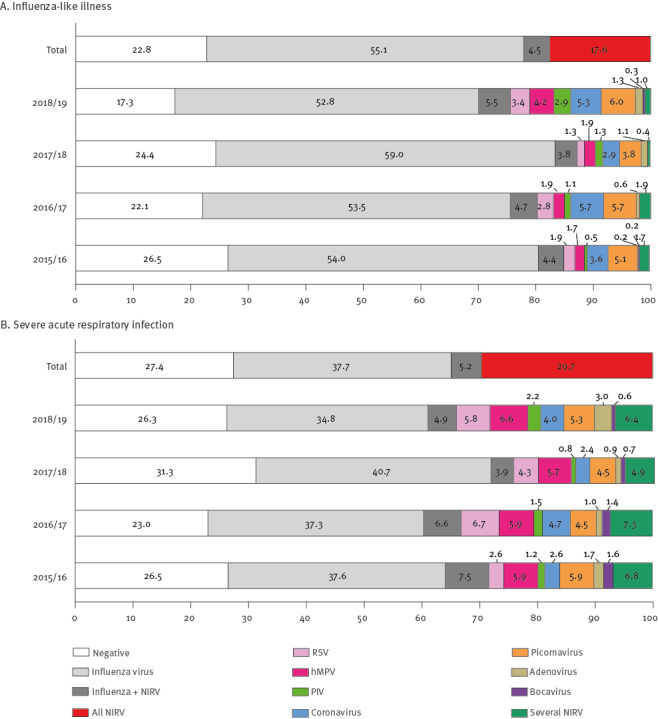
Proportions of the different respiratory viruses detected by multiplex RT-qPCR per season and overall, Belgium, 2015–2019 (n = 6,565)

Among the 1,791 ILI patients, 408 (22.8%) were negative for all respiratory viruses tested, 1,068 (59.6%) were positive for influenza virus (of which 81 were co-detections with NIRV), and 315 (17.6%) were negative for influenza virus but positive for NIRV (of which 22 were co-detections of several NIRV) (Figure 2A). Among the 4,774 SARI patients, 1,309 (27.4%) were negative for all respiratory viruses tested, 2,049 (42.9%) were positive for influenza virus (of which 250 were co-detections with NIRV) and 1,415 (29.6%) were negative for influenza virus but positive for at least one NIRV (with 291 being co-detections with several NIRV) (Figure 2B).

Coronavirus: coronavirus CoV-OC43, CoV-NL63 or CoV-229E; hMPV: human metapneumovirus; influenza virus: influenza virus type A or B; influenza + NIRV: co-detection of influenza virus with at least one non-influenza respiratory virus; negative: negative for all tested viruses; NIRV: positive for non-influenza respiratory virus(es); picornavirus: picornavirus of the *Rhinovirus* and *Enterovirus* genera or parechovirus; PIV: parainfluenzavirus type 1, 2, 3 or 4; RSV: respiratory syncytial virus type A or B; several NIRV: co-detection of several non-influenza respiratory viruses.

All seasons taken together, the most common single NIRV detections among ILI patients were picornaviruses (5.1%; 91/1,791) and coronaviruses (4.3%; 77/1,791), followed by human metapneumoviruses (hMPV) (2.3%; 42/1,791) and respiratory syncytial viruses (RSV) (2.3%; 41/1,791). Single detections of parainfluenza virus, adenovirus and bocavirus accounted for 1.4%, 0.8% and 0.1%, respectively (Supplementary Table S2). Among SARI patients, the main single NIRV detections were hMPV (6.1%; 291/4,774), RSV (5.0%; 238/4,774), picornaviruses (5.0%; 237/4,774) and coronaviruses (3.4%; 162/4,774). Single detections of parainfluenza virus, adenovirus and bocavirus accounted for 1.4%, 1.7% and 0.9%, respectively (Supplementary Table S2). Co-detections were more common among SARI patients than ILI patients. They accounted for 11.3% of the overall number of tested SARI samples (541/4,774), with influenza virus-positive (n = 250) and influenza-negative (n = 291) co-detections representing 5.2 and 6.1%, respectively.

The proportion of NIRV detection (excluding co-infections with influenza viruses) varied between 12.8% in 2017/18 and 24.3% in 2018/19 in the ILI samples (Figure 2A). In SARI patients, the proportion was higher and varied between 24.2% in 2017/18 and 33.9% in 2018/19 (Figure 2B). Within a season, the proportions of picornaviruses and coronaviruses were similar among ILI and SARI patients. On the contrary, hMPV and RSV detection rates were systematically higher in SARI than in ILI samples. Except for season 2015/16, these two viruses represented the largest groups in SARI patients, whereas picornaviruses and coronaviruses were the most frequent ones in the ILI patients. Season 2018/19 was marked by higher detection rates for parainfluenza viruses (both in ILI and SARI) and adenoviruses (in SARI) compared with previous seasons (Figure 2, Supplementary Table S2).

**Figure 3:**
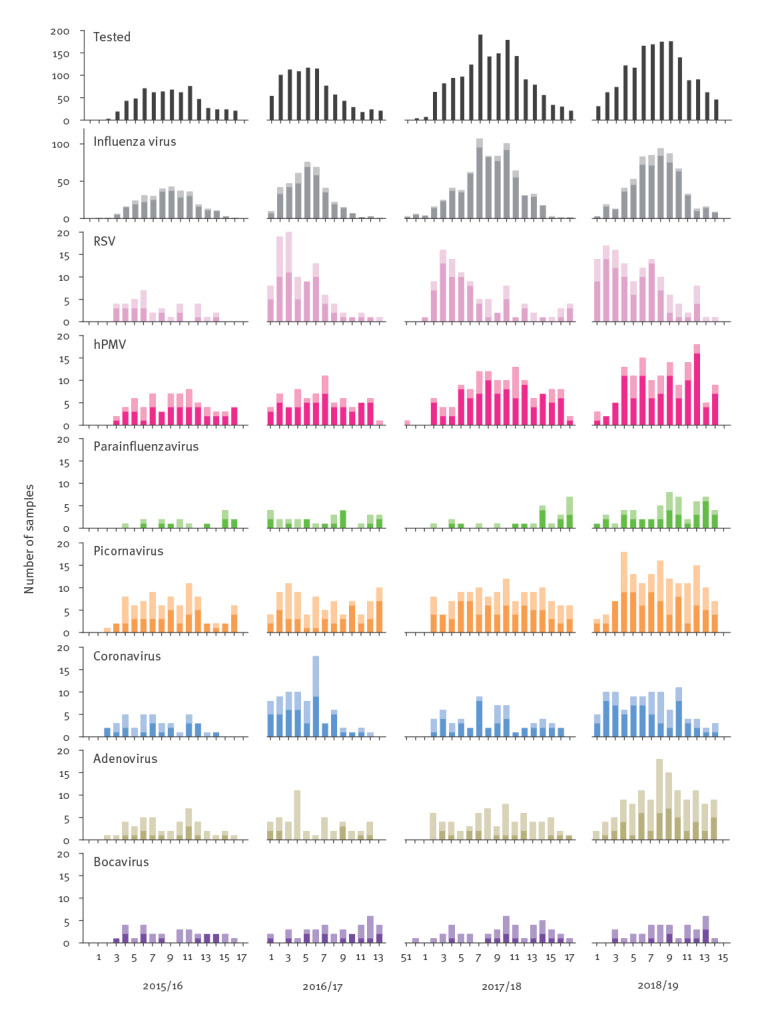
Distribution of the positive samples for different respiratory viruses by season and sampling week for patients with severe acute respiratory infection, Belgium, 2015–2019 (n = 4,774)

Both ILI and SARI surveillances were able to capture the seasonal circulation of influenza virus, but such bell-shape epidemic curves were not detected for other NIRV, with perhaps two exceptions: coronaviruses in the ILI surveillance during season 2016/17 despite the limited number of positive samples (Supplementary Figure S1) and RSV in the SARI surveillance, with the detection of the end of the RSV epidemic, which usually occurs before the influenza epidemic in western Europe [13] including in Belgium [14] (Figure 3).

hPMV: human metapneumovirus; RSV: respiratory syncytial virus.

Colour codes as in Figure 2. Solid colour: single detection; shaded colour: in co-detection with at least one other virus. The x-axes include weeks of active SARI surveillance: 2015/16, week 1 to week 17 of 2016; 2016/17, week 1 to week 13 of 2017; 2017/18, week 51 of 2017 to week 17 of 2018; 2018/19, week 1 to week 15 of 2019.

### Age-specific detection rates of non-influenza respiratory viruses and NIRV-associated SARI incidence rates

In children (younger than 15 years), the proportion of samples positive for NIRV (single or co-detection) was higher in SARI than ILI patients (Table). The proportion positive for NIRV only (excluding co-detection with influenza viruses) was 49.4% (828/1,677) in children with SARI and only 15.6% (38/244) in children with ILI (Fisher’s exact test, p < 0.001). In contrast, the proportions of positive NIRV samples among adults (15–64 years-old) and older adults (≥ 65 years-old) taken together were similar for ILI and SARI surveillance (17.9%; 267/1,488 and 18.9%; 582/3,082, respectively; Fisher’s exact test, p = 0.465).

**Table:** Respiratory virus single and co-detection in influenza-like illness and severe acute respiratory infection surveillance patients by age group, Belgium, 2015–2019 (n = 6,565)

	Children		Adults		Older adults		Missing age	Total	
	n	%	n	%	n	%	n	n	%
ILI									
Overall	244		1,364		124		59	1,791	
**Negative**	**33**	**13.5**	**327**	**24.0**	**29**	**23.4**	**19**	**408**	**22.8**
Single influenza	158	64.8	737	54.0	64	51.6	28	987	55.1
Influenza + NIRV	15	6.1	58	4.3	6	4.8	2	81	4.5
**Total influenza**	**173**	**70.9**	**795**	**58.3**	**70**	**56.5**	**30**	**1,068**	**59.6**
RSV	4	1.6	33	2.4	3	2.4	1	41	2.3
hMPV	8	3.3	30	2.2	3	2.4	1	42	2.3
Parainfluenza	3	1.2	16	1.2	4	3.2	2	25	1.4
Coronavirus	7	2.9	61	4.5	9	7.3	0	77	4.3
Picornavirus	7	2.9	79	5.8	3	2.4	2	91	5.1
Adenovirus	3	1.2	9	0.7	1	0.8	2	15	0.8
Bocavirus	0	0.0	2	0.1	0	0.0	0	2	0.1
**Total single NIRV**	**32**	**13.1**	**230**	**16.9**	**23**	**18.5**	**8**	**293**	**16.4**
NIRV coinfection	6	2.5	12	0.9	2	1.6	2	22	1.2
**Total NIRV**	**38**	**15.6**	**242**	**17.7**	**25**	**20.2**	**10**	**315**	**17.6**
SARI									
Overall	1,677		977		2,105		15	4,774	
**Negative**	**302**	**18.0**	**373**	**38.2**	**631**	**30.0**	**4**	**1,310**	**27.4**
Single influenza	407	24.3	390	39.9	996	47.3	6	1,799	37.7
Influenza + NIRV	140	8.3	32	3.3	78	3.7	0	250	5.2
**Total influenza**	**547**	**32.6**	**422**	**43.2**	**1,074**	**51.0**	**6**	**2,049**	**42.9**
RSV	105	6.3	33	3.4	100	4.8	0	238	5.0
hMPV	130	7.8	48	4.9	112	5.3	1	291	6.1
Parainfluenza	40	2.4	8	0.8	20	1.0	1	69	1.4
Coronavirus	40	2.4	39	4.0	82	3.9	1	162	3.4
Picornavirus	151	9.0	33	3.4	52	2.5	1	237	5.0
Adenovirus	63	3.8	10	1.0	10	0.5	0	83	1.7
Bocavirus	44	2.6	0	0.0	0	0.0	0	44	0.9
**Total single NIRV**	**573**	**34.2**	**171**	**17.5**	**376**	**17.9**	**4**	**1,124**	**23.5**
NIRV coinfection	255	15.2	11	1.1	24	1.1	1	291	6.1
**Total NIRV**	**828**	**49.4**	**182**	**18.6**	**400**	**19.0**	**5**	**1,415**	**29.6**

Coronavirus: coronavirus CoV-OC43, CoV-NL63 or CoV-229E; hMPV: human metapneumovirus; ILI: influenza-like illness; influenza + NIRV: co-detection of influenza virus with at least one non-influenza respiratory virus; NIRV: non-influenza respiratory virus; NIRV coinfection: co-detection of several non-influenza respiratory viruses; parainfluenza: parainfluenzavirus type 1, 2, 3 or 4; picornavirus: picornavirus of the *Rhinovirus* and *Enterovirus* genera or parechovirus; RSV: respiratory syncytial virus type A or B; SARI: severe acute respiratory infection; Single influenza: influenza virus type A or B only.

Age groups: children, < 15 years; adults, 15–64 years old; older adults, ≥ 65 years; missing, unknown age.

Percentage per column, calculated based on the overall number. Not calculated for ‘missing’ groups.

Half (82/162) of the SARI samples positive for coronavirus only were from older adults (Table). On the contrary, all SARI samples positive for bocavirus only (n = 44) and 75.9% (63/83) of those positive for adenovirus only were from children. The SARI samples positive for RSV only and hMPV only were from both children (44.1%; 105/238 and 44.7%; 130/291, respectively), mainly younger than 1 year, and from older adults (42.0%; 100/238 and 38.5%; 112/291, respectively). Picornavirus-only and parainfluenzavirus-only SARI samples were mainly from children under the age of 5 years (58.2%; 138/237 and 50.7%; 35/69, respectively, Supplementary Table S3), but also from older adults (21.9%; 52/237 and 29.0%; 20/69, respectively). Finally, SARI samples with co-detection of NIRV or of NIRV with influenza viruses were largely from children younger than 5 years (86.3%; 251/291 and 51.6%; 129/250, respectively, Supplementary Table S3)

**Figure 4:**
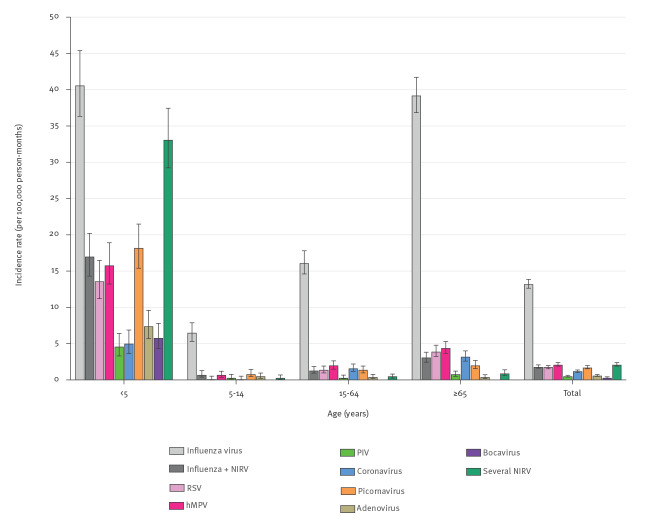
Incidence rates of virus-associated severe acute respiratory infections per 100,000 person-months by age group, Belgium, 2015–2019 (n = 4,774)

The burden of NIRV captured by influenza surveillance was partially evaluated by calculating the incidence rates of SARI associated with each virus within specific age groups and for all the samples (Figure 4 and Supplementary Table S4). NIRV incidence rates were noticeably higher in the youngest age group (< 5 years) than in the other age groups or the overall population, with RSV, hMPV and picornaviruses contributing the most as single infection (13.6, 15.8 and 18.2 per 100,000 person-months, respectively), followed by adenoviruses, bocavirus, coronaviruses and parainfluenzaviruses (7.4, 5.8, 5.0 and 4.6 per 100,000 person-months, respectively). In this age group, the incidence rate of SARI associated with all kinds of influenza-negative NIRV infections was almost double that associated with influenza (103.5 vs 57.6 per 100,000 person-months). Incidence rates of SARI associated with NIRV were low in the other age groups, in contrast to those associated with influenza (Figure 4). Despite that, the incidence rates of SARI associated with RSV, hMPV and coronaviruses in older adults were more than twice that in the overall population (3.9, 4.4 and 3.2 vs 1.8, 2.1 and 1.2 per 100,000 person-months, respectively).

Coronavirus: coronavirus CoV-OC43, CoV-NL63 or CoV-229E; hMPV: human metapneumovirus; influenza virus: influenza virus type A or B; influenza + NIRV: co-detection of influenza virus with at least one non-influenza respiratory virus; picornavirus: picornavirus of the *Rhinovirus* and *Enterovirus* genera or parechovirus; PIV: parainfluenzavirus type 1, 2, 3 or 4; RSV: respiratory syncytial virus type A or B; several NIRV: co-detection of several non-influenza respiratory viruses.

## Discussion

Here, we report on the use of several in-house multiplex qPCR assays on respiratory specimens collected through two sentinel networks of influenza surveillance (ILI and SARI) to detect a broad range of respiratory viruses of public health relevance during the winter season. Systematic testing of NIRV on samples was introduced in Belgium from the winter season 2015/16 onwards, with the use of in-house multiplex qPCRs offering the flexibility to change the panel of viruses/virus groups depending on the situation. We found that NIRV testing may explain up to one third of infections and becomes particularly useful for diagnostic purposes of paediatric SARI patients. By estimating incidence rates, we showed that NIRV even represent a higher burden than influenza viruses in the SARI patients under the age of 5 years.

A limitation of our study is that these sentinel surveillance networks collect data only during the winter season in Belgium. This is particularly true for the SARI network which operates only during the period of influenza virus activity. In addition, since the objective of the SARI surveillance is primarily to evaluate the severity of influenza viruses, the definition used might not be optimally adapted for NIRV. The criteria ‘fever’ and ‘cough’ were chosen to specifically target influenza viruses [15], but they are also characteristic of most NIRV, including RSV and hMPV [16], the two main NIRV identified in our study. Despite the differences in the case definition and the period of surveillance, our results remain comparable to those obtained in other countries regarding the NIRV identified and their proportions [17,18], but the detection rates observed here for the different NIRV might not represent their whole circulation patterns. For example, it was shown that the intensive circulation period for RSV usually precedes that of influenza viruses in western Europe [13]. Although RSV, hMPV, coronaviruses and other NIRV do not strictly follow the same pattern as influenza viruses [19], many tend to also circulate during the winter season in temperate regions. Interactions between respiratory viruses have been reported [20,21], including a negative association between rhinovirus and RSV infection in children [22]. There have also been reports of an increased risk of NIRV infections in children who received inactivated influenza vaccine in a small-scale study [23], although this was not observed in a larger study encompassing data from seven seasons [24]. Influenza sentinel surveillance thus appears as a relevant system for the study of NIRV [25,26] and we consider that extending the period of SARI surveillance in Belgium would be beneficial to better estimate their burden but also, on a long-term perspective, the potential impact of the new severe acute respiratory syndrome coronavirus 2 after the current pandemic phase, if it establishes itself as a seasonal virus.

Another limitation is that paediatric and geriatric samples are underrepresented in ILI patients, probably due to sampling bias (GPs in Belgium being less likely to have children or older people as patients or to sample them).

The systematic testing for NIRV allowed the detection of a pathogen in only one eighth of the ILI patients, suggesting that the additional effort of testing NIRV in this study population may be of limited interest and not cost-effective. In contrast, samples were NIRV-positive in almost a third of the SARI patients, in whom the morbidity and mortality associated with respiratory infection is the highest, and in almost half of the paediatric SARI patients. The burden associated with NIRV in SARI patients was age-dependent and particularly high for young children (< 5 years). While co-infections with several NIRV were rarely detected among ILI patients, they were almost as common as influenza virus infection among paediatric SARI patients younger than 5 years. The non-negligible burden associated with RSV should also be taken into consideration in the coming years, when RSV vaccines become available and vaccination is implemented [27]. This clearly shows the benefit that testing SARI patients for NIRV infections can have on the surveillance trend analysis but also, to a lesser extent, on clinical management.

Despite an important decrease in the number of specimens that were reported as negative in SARI patients following the introduction of NIRV testing, more than one fourth of SARI patients remained negative for all tested viruses. Although viruses are considered as the main cause of acute respiratory tract infections [28-30] and our methodology covers the main respiratory viruses that are involved [31], it is possible that some samples were positive for viruses that were not targeted by our multiplex qPCRs (e.g. coronavirus HKU-1, influenza C virus, cytomegalovirus, herpes simplex virus), or for bacterial or fungal respiratory pathogens, known to substantially contribute to complications in lower respiratory tract infections [32-34]. In a prospective study in 11 European countries that looked at the aetiology of lower respiratory tract infections among adults, a bacterial pathogen was detected in one fifth of the participants [35]. The difference in the proportion of ‘negative’ samples that we observed between age groups (one third among adults and one fifth among children) suggests that the pathogens causing SARI differ between children (more broadly covered by our multiplex PCRs) and adults, which could be due to the clinical signs of the SARI case definition being less specific for adults, especially older adults [36,37].

## Conclusion

Our study demonstrates the added value of testing NIRV with a flexible method and confirms that the burden of disease associated with NIRV in SARI patients should not be underestimated, especially in children under the age of 5 years for whom it is even higher than that of influenza viruses. Although more expensive than other surveillance systems, SARI sentinel surveillance offers the advantage that it relies on a precise case definition and collects data on complications and co-morbidities. Throughout the years, as data obtained in a standardised manner accumulate, such system could provide better estimates of the burden of NIRV.

## Supplementary Data

211000 Supplement
