# Supplementary material for: Spotlight influenza: Extending influenza surveillance to detect non-influenza respiratory viruses of public health relevance: analysis of surveillance data, Belgium, 2015 to 2019
Source: Euro Surveill. 2021 Sep 23;26(38):2001104. doi: 10.2807/1560-7917.ES.2021.26.38.2001104 (PMC8462033; doi:10.2807/1560-7917.ES.2021.26.38.2001104)
Supplement: Supplement [file 20-01104_BARBEZANGE_Supplement.pdf]

This supplementary material is hosted by Eurosurveillance as supporting information alongside the article "Extending influenza surveillance to detect non-influenza respiratory viruses of public health relevance: analysis of surveillance data, Belgium, 2015 to 2019", on behalf of the authors who remain responsible for the accuracy and appropriateness of the content. The same standards for ethics, copyright, attributions and permissions as for the article apply. Supplements are not edited by Eurosurveillance and the journal is not responsible for the maintenance of any links or email addresses provided therein.

Supplementary Table S1: Characteristics of influenza-like illness and severe acute respiratory infection patients for influenza seasons, Belgium, 2015-2019.

	ILI		SARI per hospital and total													
			Site 1		Site 2		Site 3		Site 4		Site 5		Site 6		total	
	n	%	n	%	n	%	n	%	n	%	n	%	n	%	n	%
Overall	1791		1071		514		491		832		1034		832		4774	
Age group																
<1	6	0.3	107	10.0	246	47.9	44	8.9	110	13.2	86	8.3	160	19.2	753	15.8
1-4	41	2.3	99	9.2	135	26.3	40	8.2	84	10.1	190	18.4	163	19.6	711	14.9
5-14	197	11.0	27	2.5	28	5.5	5	1.0	35	4.2	67	6.5	63	7.6	225	4.7
15-64	1365	76.2	245	22.9	61	11.9	141	28.7	204	24.5	165	16.0	158	19.0	974	20.4
65-84	112	6.3	425	39.7	33	6.4	211	43.0	288	34.5	344	33.3	208	25.0	1509	31.6
≥85	12	0.7	166	15.5	11	2.1	47	9.6	111	13.3	182	17.6	73	8.8	590	12.4
Missing	57	-	2	-	0	-	3	-	0	-	0	-	7	-	12	-
Sex																
Female	833	49.2	526	48.3	238	46.8	176	44.1	391	47.5	529	51.4	372	45.6	2222	47.8
Male	859	50.8	552	51.7	271	53.2	223	55.9	433	52.5	501	48.6	448	54.6	2428	52.2
Missing	99	-	3	-	5	-	92	-	8	-	4	-	12	-	124	-
Season																
2015/16	411	22.9	94	8.8	119	23.2	73	14.9	81	9.7	190	18.4	134	16.1	691	14.5
2016/17	474	26.5	255	23.8	99	19.3	96	19.6	83	10.0	223	21.6	156	18.8	912	19.1
2017/18	525	29.3	363	33.9	177	34.3	156	31.8	311	37.4	317	30.7	287	34.5	1611	33.8
2018/19	281	15.7	359	33.5	119	23.2	166	33.8	357	42.9	304	29.4	255	30.7	1560	32.7

Age groups: <1, under 1 year old; 1-4, from 1 to below 5 years old; 5-14, from 5 to below 15 years old; 15-64, from 15 to below 65 years old; 65-84, from 65 to below 85 years old; ≥85, above 85 years old; unk, unknown age. Percentage per column, calculated based on overall. Not calculated for 'missing' groups.

ILI: influenza-like illness; SARI: severe acute respiratory infection

Supplementary Table S2: Test results from the respiratory virus panel for influenza-like illness and severe acute respiratory infection surveillance samples per season, Belgium, 2015-2019.

	2015/16		2016/17		2017/18		2018/19		total	
	n	%	n	%	n	%	n	%	n	%
ILI										
overall	411	-	474	-	525	-	381	-	1791	-
negative	109	26.5	105	22.1	128	24.2	66	17.3	408	22.8
Single influenza	222	54.0	254	53.5	310	59.0	201	62.8	987	55.1
Influenza + NIRV	18	4.4	22	4.7	20	3.8	21	5.5	81	4.5
Total influenza	240	58.4	276	58.2	330	62.9	222	58.3	1068	59.6
RSV	8	1.9	13	2.8	7	1.3	13	3.4	41	2.3
hMPV	7	1.7	9	1.9	10	1.9	16	4.2	42	2.3
parainfluenza	2	0.5	5	1.1	7	1.3	11	2.9	25	1.4
coronavirus	15	3.6	27	5.7	15	2.9	20	5.3	77	4.3
picornavirus	21	5.1	27	5.7	20	3.8	23	6.0	91	5.1
adenovirus	1	0.2	3	0.6	6	1.1	5	1.3	15	0.8
bocavirus	1	0.2	0	0.0	0	0.0	1	0.3	2	0.1
Total single NIRV	55	13.4	84	17.8	65	12.4	89	23.4	293	16.4
NIRV coinfection	7	1.7	9	1.9	2	0.4	4	1.0	22	1.2
SARI										
overall	691	-	912	-	1611	-	1560	-	4774	-
negative	183	26.5	210	23.0	504	31.3	411	26.3	1309	27.4
Single influenza	260	37.6	340	37.3	655	40.7	543	34.8	1799	37.7
Influenza + NIRV	52	7.5	60	6.6	62	3.9	76	4.9	250	5.2
Total influenza	312	45.2	400	43.9	717	44.5	619	39.7	2049	42.9
RSV	18	2.6	61	6.7	69	4.3	91	5.8	238	5.0
hMPV	41	5.9	54	5.9	92	5.7	104	6.6	291	6.1
parainfluenza	8	1.2	14	1.5	13	0.8	34	2.2	69	1.4
coronavirus	18	2.6	43	4.7	39	2.4	62	4.0	162	3.4
picornavirus	41	5.9	41	4.5	72	4.5	83	5.3	237	5.0
adenovirus	12	1.7	9	1.0	15	0.9	47	3.0	83	1.7
bocavirus	11	1.6	13	1.4	11	0.7	9	0.6	44	0.9
Total single NIRV	149	21.6	235	25.9	311	19.3	430	27.5	1124	23.6
NIRV coinfection	47	6.8	67	7.3	78	4.9	100	6.4	291	6.1

Percentage per column, calculated based on overall for each surveillance network. Not calculated for 'missing' groups.

ILI: influenza-like illness; SARI: severe acute respiratory infection

Supplementary Table S3: Test results from the respiratory virus panel for severe acute respiratory infection surveillance samples per age group, Belgium, 2015-2019.

	<1		1-4		5-14		15-64		65-84		≥85		Missing		total	
	n	%	n	%	n	%	n	%	n	%	n	%	n	%	n	%
overall	748	-	706	-	223	-	977	-	1512	-	593	-	15	-	4774	-
**negative**	**129**	**17.2**	**103**	**14.6**	**70**	**31.4**	**373**	**38.2**	**478**	**31.6**	**153**	**25.8**	**4**	**-**	**1310**	**27.4**
Single influenza	107	14.3	201	28.5	99	44.4	390	39.9	679	44.9	317	53.5	6	-	1799	37.7
Influenza + NIRV	45	6.0	84	11.9	11	4.9	32	3.3	52	3.4	26	4.4	0	-	250	5.2
**Total influenza**	**152**	**20.3**	**285**	**40.4**	**110**	**49.3**	**422**	**43.2**	**731**	**48.3**	**343**	**57.8**	**6**	**-**	**2049**	**42.9**
RSV	84	11.2	19	2.7	2	0.9	33	3.4	70	4.6	30	5.1	0	-	238	5.0
hMPV	68	9.1	52	7.4	10	4.5	48	4.9	81	5.4	31	5.2	1	-	291	6.1
parainfluenza	21	2.8	14	2.0	5	2.2	8	0.8	18	1.2	2	0.3	1	-	69	1.4
coronavirus	31	4.1	7	1.0	2	0.9	39	4.0	67	4.4	15	2.5	1	-	162	3.4
picornavirus	82	11.0	56	7.9	13	5.8	33	3.4	41	2.7	11	1.9	1	-	237	5.0
adenovirus	23	3.1	33	4.7	7	3.1	10	1.0	8	0.5	2	0.3	0	-	83	1.7
bocavirus	17	2.3	27	3.8	0	0.0	0	0.0	0	0.0	0	0.0	0	-	44	0.9
**Total single NIRV**	**326**	**43.6**	**208**	**29.5**	**39**	**17.5**	**171**	**17.5**	**285**	**18.8**	**91**	**15.3**	**4**	**-**	**1124**	**23.5**
NIRV coinfection	141	18.9	110	15.6	4	1.8	11	1.1	18	1.2	6	1.0	1	-	291	6.1
**Total NIRV**	**467**	**62.4**	**318**	**45.0**	**43**	**19.3**	**182**	**18.6**	**303**	**20.0**	**97**	**16.4**	**5**	**-**	**1415**	**29.6**

NIRV: non-influenza respiratory virus

Age groups: <1, under 1 year old; 1-4, from 1 to below 5 years old; 5-14, from 5 to below 15 years old; 15-64, from 15 to below 65 years old; 65-84, from 65 to below 85 years old; ≥85, above 85 years old; missing, unknown age.

Percentage per column, calculated based on overall. Not calculated for 'missing' groups.

Supplementary Table S4: Incidence rates of virus-associated severe acute respiratory infections per 100,000 person-months by age group, Belgium, 2015-2019.

	<5			5-14			15-64			≥65			All samples		
	IR	Lower	Upper	IR	Lower	Upper	IR	Lower	Upper	IR	Lower	Upper	IR	Lower	Upper
Single influenza	40.6	36.3	45.4	6.5	5.3	7.9	16.1	14.6	17.8	39.2	36.8	41.7	13.2	12.6	13.9
Influenza + NIRV	17.0	14.3	20.2	0.7	0.4	1.3	1.3	0.9	1.9	3.1	2.5	3.8	1.8	1.6	2.1
RSV	13.6	11.2	16.5	0.1	0.0	0.5	1.4	1.0	1.9	3.9	3.2	4.8	1.8	1.5	2.0
hMPV	15.8	13.2	18.9	0.7	0.4	1.2	2.0	1.5	2.6	4.4	3.7	5.3	2.1	1.9	2.4
coronavirus	5.0	3.6	6.9	0.1	0.0	0.5	1.6	1.2	2.2	3.2	2.6	4.0	1.2	1.0	1.4
picornavirus	18.2	15.4	21.5	0.8	0.5	1.5	1.4	1.0	1.9	2.0	1.6	2.7	1.7	1.5	2.0
parainfluenzavirus	4.6	3.3	6.4	0.3	0.1	0.8	0.3	0.2	0.7	0.8	0.5	1.2	0.5	0.4	0.6
bocavirus	5.8	4.3	7.8	0.0	-	-	0.0	-	-	0.0	-	-	0.3	0.2	0.4
adenovirus	7.4	5.7	9.6	0.5	0.2	1.0	0.4	0.2	0.8	0.4	0.2	0.7	0.6	0.5	0.8
NIRV coinfection	33.1	29.2	37.4	0.3	0.1	0.7	0.5	0.3	0.8	0.9	0.6	1.4	2.1	1.9	2.4

NIRV: non-influenza respiratory virus

Age groups: <5, under 5 years old; 5-14, from 5 to below 15 years old; 15-64, from 15 to below 65 years old; ≥65, above 65 years old.

IR, incidence rate; Lower and upper bounds of 95% confidence interval of IR

Supplement Figure S1: Distribution of the positive samples for each respiratory virus by season and sampling week for ILI patients. Colour codes: black, tested by multiplex RT-qPCRs (Tested); light grey, influenza virus type A or B (Influenza); light pink, Respiratory Syncytial virus type A or B (RSV); fuchsia, human metapneumovirus (hMPV); green, parainfluenzavirus type 1, 2, 3 or 4 (PIV); orange, picornavirus of the *rhinovirus* and *enterovirus* genera or parechovirus (Pico); blue, coronavirus CoV-OC43, CoV-NL63 or CoV-229E (Corona); light brown, adenovirus (Adeno); purple, bocavirus (Boca). Plain bar, single detection; chequered pattern, in co-detection with at least one other virus. X-axis, weeks of active ILI surveillance: week 40 of year Y to week 20 of year Y+1.

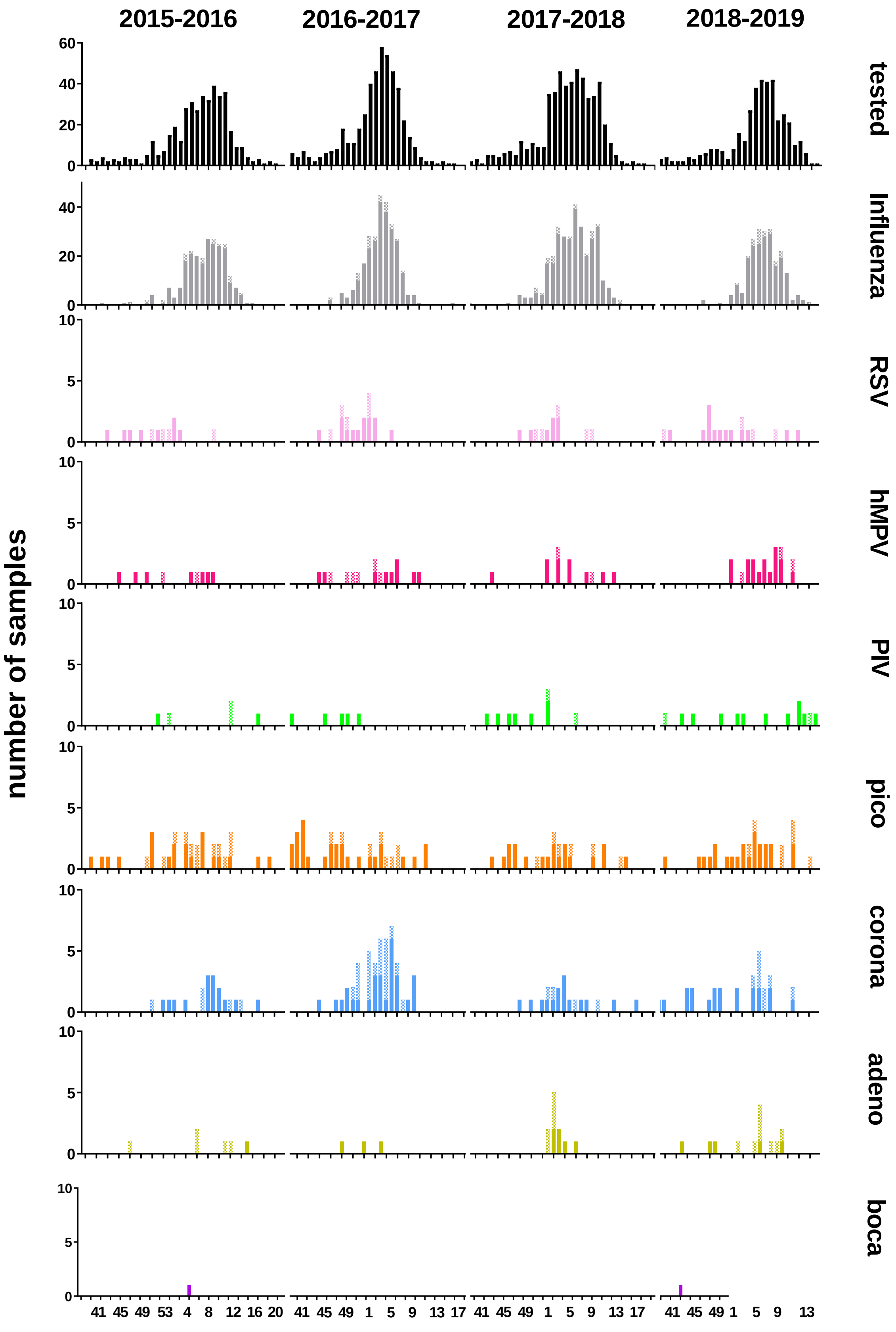

2015-2016
2016-2017
2017-2018
2018-2019
number of samples
tested
Influenza
RSV
hMPV
PIV
pico
corona
adeno
boca
0
5
10
20
40
60
41 45 49 53 4 8 12 16 20
41 45 49 1 5 9 13 17
41 45 49 1 5 9 13 17
41 45 49 1 5 9 13
